# Duodenal perforation by an inferior vena cava filter with staphylococcal bacteremia: a case report

**DOI:** 10.1186/s13256-016-0901-z

**Published:** 2016-05-04

**Authors:** Sunil Pokharel, Catherine Bartholomew, Zing Zau

**Affiliations:** 1 Department of Medicine, Albany Medical Center, 47 New Scotland Avenue, Mail Code 17, Albany, NY 12208 USA; 2 Department of Gastroenterology, Albany Medical Center, 47 New Scotland Avenue, Mail Code 48, Albany, NY 12208 USA

**Keywords:** Perforation, Bacteremia, Inferior vena cava

## Abstract

**Background:**

Inferior vena cava filter complications can range from dislodgement to perforation. Patients who present with concomitant bacteremia have rarely been reported. Persistent bacteremia usually results from direct bacterial seeding from a source other than perforation of surrounding viscus. It is unclear if the risk of perforation is higher in patients who are bacteremic due to other causes.

**Case presentation:**

We report an interesting case of a 67-year-old white woman who presented with fever, chills, and right upper quadrant abdominal pain. Her blood cultures were positive for methicillin-sensitive *Staphylococcus aureus* with no obvious source. Upon further investigation, she was found to have an inferior vena cava filter perforating her duodenum. The cause of her abdominal pain was explained by the inferior vena cava filter penetrating the duodenum; however, the source of bacteremia could not be ascertained. The inferior vena cava filter was removed successfully, and she was discharged on an intravenous antibiotic. Her symptoms resolved soon after the filter was removed.

**Conclusions:**

The use of inferior vena cava filters has increased significantly in recent years. This is likely due to their wider availability and safer placement techniques. With increasing use, the complications arising from these filters have been on the rise as well. It is very important for clinicians to be aware of these complications to avoid delays in diagnosis and patient care.

## Background

Inferior vena cava (IVC) filters are commonly used for the prevention of pulmonary embolisms when anticoagulation is contraindicated or ineffective [1, 2]. Duodenal perforation has been reported as one of the late complications of IVC filter placement. It is exceedingly rare, with only 25 cases reported in a systemic review published in 2012 [3]. We report a unique case of a patient with duodenal perforation by an IVC filter with concomitant methicillin-sensitive staphylococcal bacteremia.

## Case presentation

**Fig. 1:**
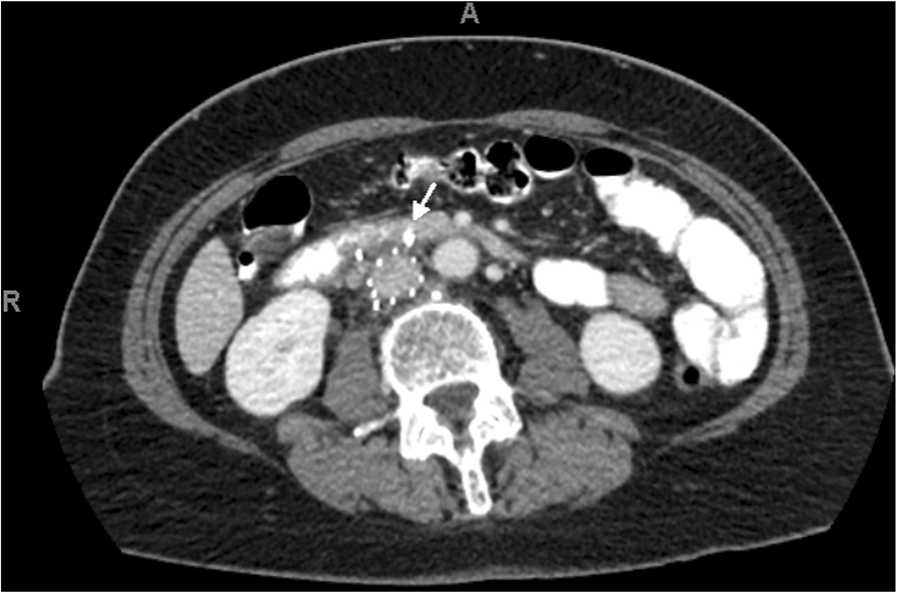
Computed tomography scan Abdomen (axial) showing struts of filter extending to adjacent structures after penetrating the wall of inferior vena cava. Arrow pointing to one of the anterior struts of inferior vena cava filter penetrating the duodenum

**Fig. 2:**
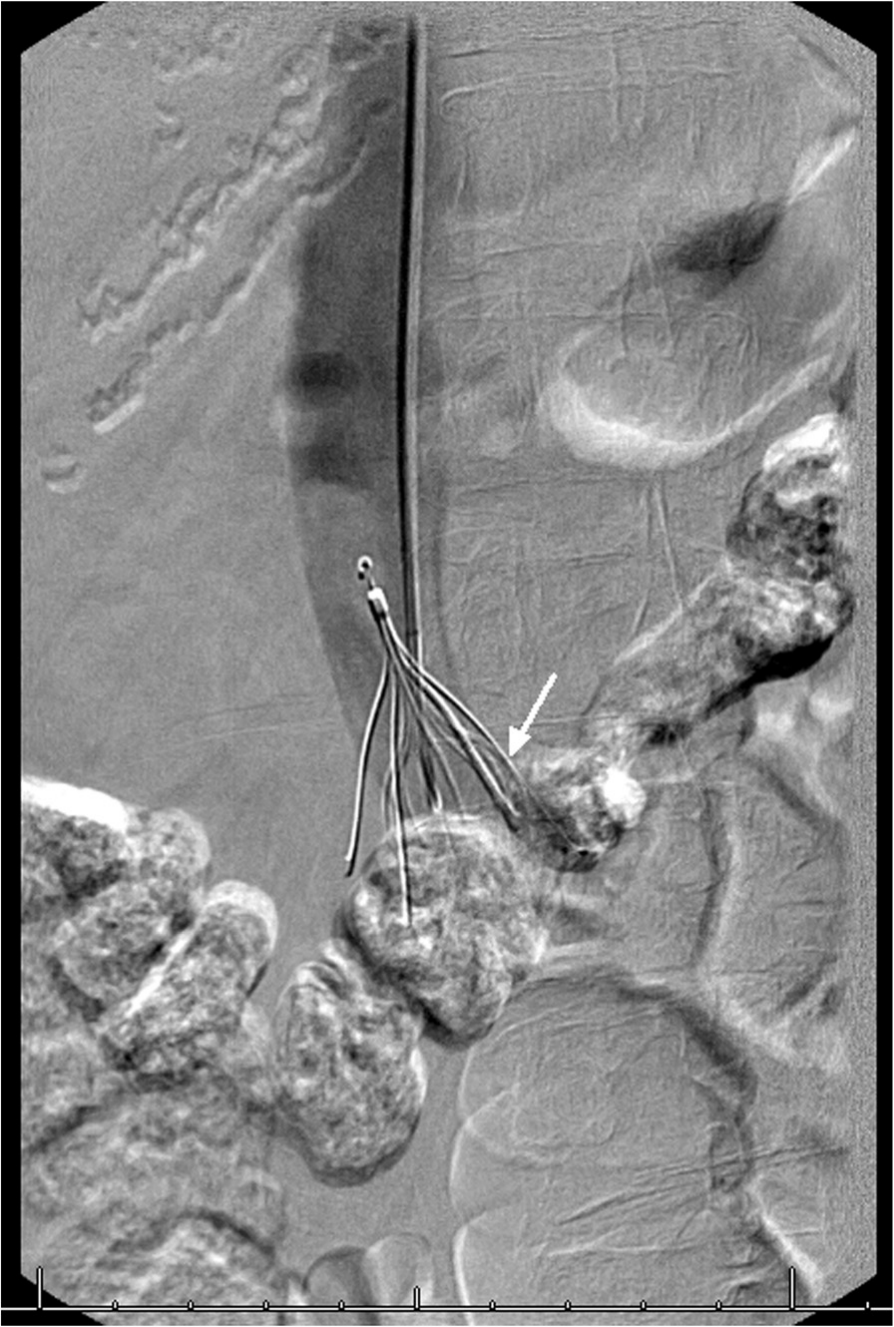
Struts of filter penetrating into the lumen of third portion of duodenum. Arrow pointing to the struts of inferior vena cava filter entering the third portion of duodenum

Approximately 6 months before presentation to our institution, a 67-year-old white woman had undergone placement of an IVC filter for bilateral pulmonary embolism after anticoagulation was contraindicated due to groin hematoma. She presented to our hospital with a 2-day history of fever, chills, and right upper quadrant abdominal pain. Her physical examination was normal except for mild right upper quadrant tenderness. Her blood cultures came back positive for methicillin-sensitive *Staphylococcus aureus*. No external source of infection was found during a detailed physical examination. She had intact skin with no ulcers or abscesses. She denied ever using intravenous drugs. The results of echocardiography were normal with no valvular vegetation. A computed tomographic scan of her abdomen and pelvis was ordered to rule out an intraabdominal infection source. Interestingly, the scan showed four struts of filter penetrating the wall of the IVC by approximately 1.5 cm. The anterior two struts extended into the lumen of the third portion of the duodenum, with the rest extending into the retroperitoneum, abutting the third lumbar vertebra and right ureter (as shown in Figs. 1 and 2). The gastroenterology team deferred upper endoscopy due to risk of barotrauma.

The cause of the patient’s abdominal pain was explained by the IVC filter penetrating the duodenum; however, the source of bacteremia could not be ascertained. The duodenum is a sterile part of the gastrointestinal tract and was unlikely to be the source of bacteremia. It was unclear if the bacteremia was a mere coincidence or whether it contributed to this complication by any means. Hopefully, more cases such as this one will clarify this in the future. A vascular surgeon and an interventional radiologist were consulted. The patient underwent successful retrieval of the IVC filter using a superior vena caval approach with a minimally invasive technique. She experienced no complications. She was discharged on an intravenous antibiotic. Anticoagulation was resumed upon discharge.

## Discussion

An IVC filter provides a mechanical barrier that prevents pulmonary embolisms originating in the veins of the lower extremities, pelvis, and IVC [1]. The indications for filter placement include patients with venous thromboembolism who have contraindications to anticoagulation, those with recurrent pulmonary embolisms while being adequately anticoagulated, and patients who develop complications due to anticoagulation [1, 2]. IVC filters have been used since the early 1970s for the treatment of venous thromboembolic disease, and retrievable filters have been used increasingly since their introduction in 2001 [4].

Permanent filters cannot be removed or repositioned. Retrieval of the temporary IVC filter is often straightforward and can be done with a high degree of success, ranging from 93 % to 100 % [5, 6]. Removal within 30 days is typical, but successful filter removal more than 1 year after implant has been reported. Kwok *et al.* described a combined jugular and femoral approach for retrieving an embedded filter [7].

Caval perforation is a well-known complication of various types of IVC filters, and, while it occurs up to 40 % of cases, it is thought to be largely asymptomatic [8–10]. Multiple case reports highlight the range of potential complications once the integrity of the vena cava has been breached. These complications include upper and lower gastrointestinal bleed, aortic and vertebral erosion, ureteric erosion, and aortoduodenal fistula [1, 3, 4, 9–11]. However, to date, only one case of staphylococcal bacteremia secondary to an infected IVC filter has been reported. This case occurred in an intravenous drug user who presented with multiple abscesses [11]. Despite the obvious benefits of retrievable filters, studies unfortunately suggest that the retrieval rates of temporary or retrievable filters are quite low and seldom exceed 20 % in most series [1, 2, 5].

## Conclusions

Duodenal perforation by an IVC filter is an uncommon complication. The concomitant bacteremia found in our patient was more likely coincidental than causal. Once a patient is persistently bacteremic, there is a significant risk of the IVC filter becoming infected. It should be taken out unless there is an absolute contraindication not to do so. It is unclear if bacteremia from another source will make someone vulnerable to this complication. The incidence of duodenal perforation is rising with the increasing use of IVC filters and more readily available diagnostic tools. Physicians should have a high degree of clinical suspicion in someone presenting with persistent abdominal pain despite normal routine evaluations.

## Consent

Written informed consent was obtained from the patient for publication of this case report and any accompanying images. A copy of the written consent is available for review by the Editor-in-Chief of this journal.
