# Erratum to: Duodenal perforation by an inferior vena cava filter with staphylococcal bacteremia: a case report

**DOI:** 10.1186/s13256-016-0954-z

**Published:** 2016-06-23

**Authors:** Sunil Pokharel, Catherine Bartholomew, Zhi Alan Cheng

**Affiliations:** 1 Department of Medicine, Albany Medical Center, 47 New Scotland Avenue, Mail Code 17, Albany, NY 12208 USA; 2 Department of Gastroenterology, Albany Medical Center, 47 New Scotland Avenue, Mail Code 48, Albany, NY 12208 USA

## Erratum

Unfortunately, the original version of this article [1] contained an error. The third author’s surname was incorrect. The corrected author name can be found below. The author’s name has been corrected in the original article and is also included correctly below.

Zhi Alan Cheng is the correct name.
